# Mosquito feeding patterns in the context of West Nile and eastern equine encephalitis viruses in eastern Ontario, Canada

**DOI:** 10.1111/mve.70077

**Published:** 2026-05-14

**Authors:** Colton R. A. Stephens, Mark R. Forbes, Scott Wilson, Antoinette Ludwig, David R. Lapen, Greg W. Mitchell

**Affiliations:** ^1^ Department of Biology Carleton University Ottawa Ontario Canada; ^2^ Wildlife Research Division Environment and Climate Change Canada Delta British Columbia Canada; ^3^ Public Health Risk Sciences Division National Microbiology Laboratory, Public Health Agency of Canada St‐Hyacinthe Quebec Canada; ^4^ Groupe de recherche en épidémiologie des zoonoses et santé publique, Faculté de médecine vétérinaire Université de Montréal St‐Hyacinthe Quebec Canada; ^5^ Science and Technology Branch Agriculture and Agri‐Food Ottawa Ontario Canada; ^6^ Wildlife Research Division Environment and Climate Change Canada Ottawa Ontario Canada

**Keywords:** Bloodmeal host, Bridge mosquito vector, Eastern equine encephalitis virus, Enzootic mosquito vector, West Nile virus

## Abstract

West Nile virus (WNV) and eastern equine encephalitis virus (EEEV) are typically maintained via enzootic cycles between birds and mosquitoes (Diptera: Culicidae). In North America, the blood hosts of mosquitoes involved in pathogen circulation are poorly described for many regions, limiting our ability to predict disease outbreaks. Our objectives were to (i) identify local (Eastern Ontario, Canada) bloodmeal hosts fed on by mosquitoes using DNA barcoding and (ii) investigate if the bloodmeal hosts identified were representative of the entire host community using species accumulation curves and Chao richness estimates. We successfully identified 313 bloodmeal hosts from 292 mosquitoes belonging to the species *Aedes cinereus*, *Aedes vexans*, *Coquillettidia perturbans*, *Culex pipiens/restuans, Ochlerotatus stimulans* and *Ochlerotatus trivittatus*, with 257 and 56 bloodmeal hosts identified at the species and genus levels, respectively. Concurrent with the literature, *Culex* species almost exclusively fed on birds, *C. perturbans* fed on a mixture of birds and mammals, and the remaining mosquito species fed almost exclusively on mammals. Eastern cottontail rabbits (*Sylvilagus floridanus*) were the most common mammal species fed on, driven by *A. vexans* and *O. trivittatus*. With respect to WNV and EEEV enzootic cycles, *Culex* species, the main vectors for WNV transmission, were observed to forage on American robins (*Turdus migratorius*), an amplification host for WNV, highlighting a pathway by which WNV could be maintained in our study region. *A. vexans*, a potential bridge vector, was also observed to forage on a relatively high frequency of humans (*Homo sapiens*), suggesting a pathway by which humans could be exposed to WNV. *C. perturbans*, a potential bridge vector for EEEV transmission, was also observed to forage on both an American Robin (also an amplifying host for EEEV) as well as on humans in a relatively high frequency, illustrating a potential pathway by which EEEV exposure in humans could occur. Our species accumulation curves and Chao estimates of richness with respect to bloodmeal hosts, indicated that the bloodmeal host community was not completely characterized. Together, our results contribute to a better understanding of mosquito bloodmeal hosts in eastern North America and suggest a larger or more directed sampling effort would be needed to characterize the entire bloodmeal host community in our study region.

## Graphical abstract

- Mosquitoes in Eastern Ontario, Canada, readily fed on bloodmeal hosts consistent with patterns observed in other regions of North America.
- Enzootic and bridge vector mosquitoes fed on amplification hosts like American Robins (*Turdus migratorius*) together with humans, highlighting a potential route for WNV and EEEV transmission to human populations.
- Our sampling effort as determined by species accumulation curves and Chao richness estimates indicated these results are not fully representative of their diet.

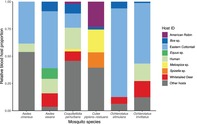

## BACKGROUND

West Nile virus (WNV; family: Flaviviridae) and eastern equine encephalitis virus (EEEV; family: Togaviridae) are two arboviruses that infect people, livestock and wildlife in North America (Chancey et al., 2015, Armstrong and Andreadis 2022). Both viruses are typically maintained through transmission and amplification cycles between birds and mosquitoes (Diptera: Culicidae), with occasional spillover to other species like humans (*Homo sapiens*) and horses (*Equus caballus*; Davis et al., 2008). A surprising number of animal species are susceptible to infection by these viruses, which can lead to serious and often fatal neurological disease (Marra et al., 2004). In the USA, over 7 million people have likely been infected with WNV since its introduction to the country in 1999 (Ronca et al., 2021), with 60,607 known cases as of 2024 and 2958 deaths as of 2023 (CDC, 2022). In Canada, a total of 4874 known human cases were recorded between 2003 and 2024 (Public Health Agency of Canada, 2022). Throughout North America, over 15 million birds are estimated to have died from the virus (George et al., 2015). Outside of these countries, WNV has now been found in all continents except Antarctica (Reisen, 2013). Whereas WNV was introduced to North America (Lanciotti et al., 1999), EEEV is endemic to the continent (Brault et al., 1999). In the United States, EEEV is regularly detected in eastern regions including the Gulf of Mexico, south of the Great Lakes and states along the east coast (CDC, n.d.). In Canada, fewer than 5 human cases of EEEV have been reported in Ontario and Quebec, with one death (Public Health Agency of Canada, 2022). A total of 38 equine cases have also been recorded, including 23 in Ontario, 14 in Quebec and 1 in Nova Scotia, Canada (Public Health Agency of Canada, 2022). While not as common as WNV, EEEV typically results in more severe infections with higher fatality rates, that is, 90% mortality rate for unvaccinated horses and 30% for humans if neuroinvasive disease develops (Armstrong & Andreadis, 2022; Public Health Agency of Canada, 2011). In Canada, the prevalence of both viruses are expected to increase as a result of climate change creating new mosquito habitats and extending the pathogen transmission season (Ludwig et al., 2019).

In North America, *Culex* species are often implicated as the main vectors of WNV transmission (Andreadis, 2012) and *Culiseta melanura* is implicated as the primary vector for EEEV (Armstrong & Andreadis, 2010), due to their vectorial capacities and feeding preferences for bloodmeal hosts (i.e., the species they prefer to feed on) that support high viral loads. However, other mosquito species can function in supplementary roles (Armstrong & Andreadis, 2010; Pérez‐Ramírez et al., 2014; Sardelis, 2001; Tiawsirisup et al., 2008; Tiawsirisup, Platt, Evans, & Rowley, 2005; Turell et al., 2000, 2001, 2005). For example, *Aedes vexans* and *Coquillettidia perturbans* and can function as bridge vectors (i.e., species that transmit pathogens between amplifying hosts and dead‐end hosts) between birds and humans for EEEV and WNV, respectively (Burkett‐Cadena et al., 2022; Tiawsirisup et al., 2008). A critical first step to understanding how these viruses continue to persist and threaten North American human, livestock and wildlife populations, is understanding mosquito feeding patterns in relation to their bloodmeal hosts. Once bloodmeal host species are identified, follow‐up studies can be carried out to understand their specific role in pathogen transmission cycles.

In North America, our current understanding of mosquito diets is lacking in many regions and is complicated by the complexity of mosquito feeding behaviours. Some mosquito species feed preferentially on specific animals or broader animal groups like birds or mammals (Wolff & Riffell, 2018). Other mosquito species do not have strong feeding preferences and will feed opportunistically on whichever animals are readily accessible to them. However, even mosquitoes with strong diet preferences can feed on other species during periods where their preferred bloodmeal hosts are scarce or absent (Lyimo & Ferguson, 2009; Wolff & Riffell, 2018). Notably, mosquitoes with strong feeding preferences for birds can readily utilize mammals during migratory periods when fewer birds are available to them (Thiemann et al., 2011). This host switching has also been implicated as the cause of some human disease outbreaks after mosquitoes switched to feeding on humans during migratory periods when avian densities decreased (Kilpatrick, Kramer, et al., 2006). Last, species‐specific mosquito diets can also vary across populations owing to local genetic adaptation and divergence (Lyimo & Ferguson, 2009; Takken & Verhulst, 2013; Wolff & Riffell, 2018). Together, these studies suggest that understanding virus transmission pathways via bloodmeal hosts is arguably best achieved at a regional level, rather than generalized across mosquito species and disparate geographic areas.

One specific tool to help identify regionally specific pools of bloodmeal hosts is DNA barcoding of mosquito bloodmeals (Hernández‐Triana et al., 2017; Leta et al., 2025; Reeves et al., 2018). While the frequency of bloodmeal hosts identified through barcoding is not necessarily indicative of preferences, given the availability or abundance of bloodmeal hosts is rarely known, it can begin to illustrate the broader ecological community that may be involved in regional pathogen dynamics and provides important information for targeted future sampling. With respect to the latter, DNA barcoding of mosquito bloodmeals can also be used to derive bloodmeal host species accumulation curves that can in turn be used to plan future sampling efforts (Lee et al., 2025; Ortiz et al., 2025).

Given that our understanding of mosquito bloodmeal hosts across mosquito species is limited in Canada and many other areas of North America, the goal of this study is to characterize the bloodmeal host community in Eastern Ontario, Canada, that could potentially be involved in WNV and EEEV transmission. Specifically, our first objective is to determine and differentiate the animal species fed on by mosquitoes in our study region using DNA barcoding. Our second objective is to assess how adequately the bloodmeal host community identified here is reflective of the true range of species fed on by local mosquitoes. It is our hope that this study will help inform future research efforts to better understand WNV and EEEV dynamics in temperate eastern North America.

## MATERIALS AND METHODS

### Study sites

**FIGURE 1:**
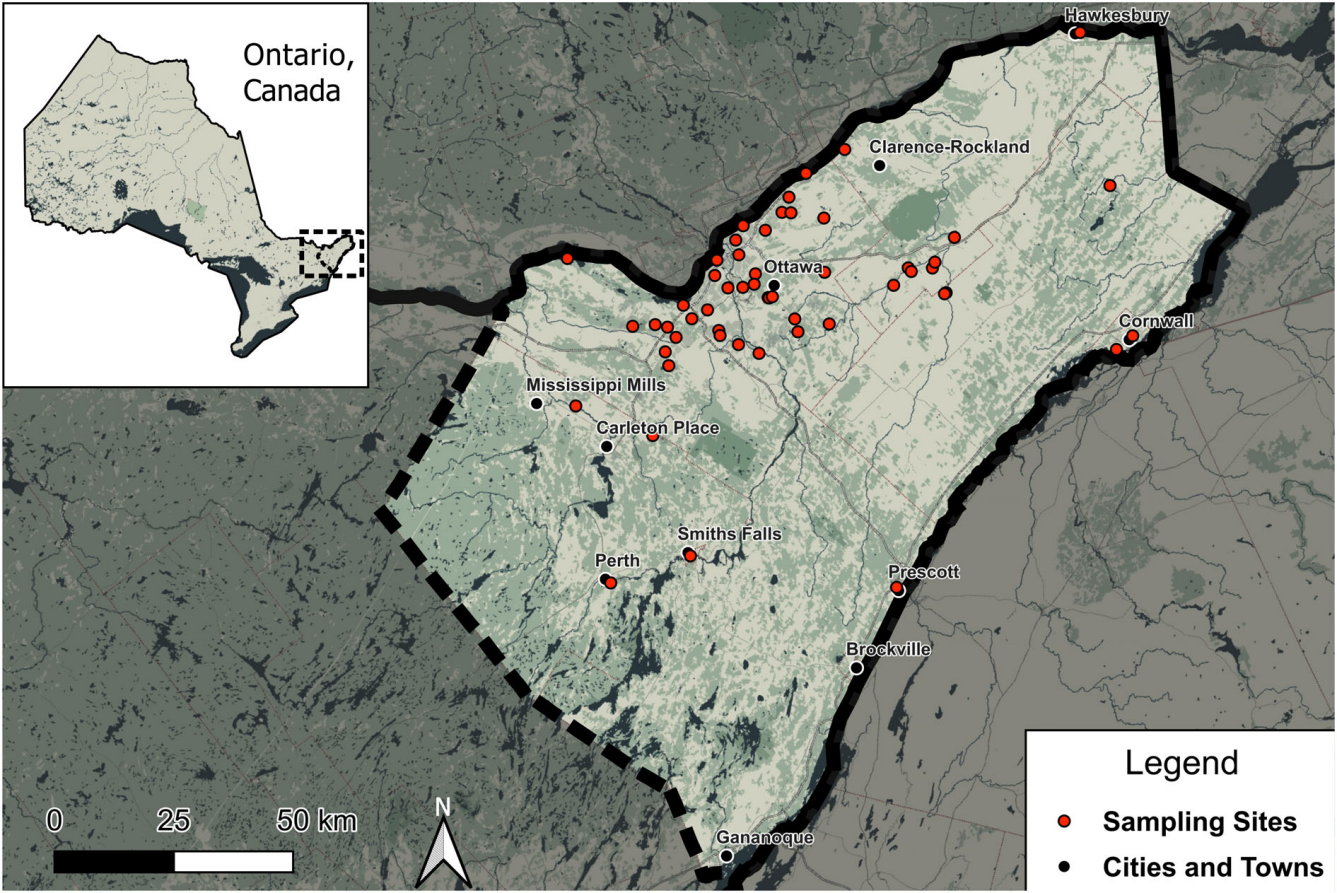
Mosquito sampling locations in Eastern Ontario, Canada. Main study area (approximate centre of study area: 45.07748706608795, −75.63924880018072) is represented by highlighted area. Inset: Province of Ontario, Canada with study region indicated by box with hatched line. Red dots in main figure represent sampling sites. Black dots represent cities and towns.

Research took place in Eastern Ontario, Canada, which is a fragmented mosaic of agricultural cropland, urban environments, rivers, mixed deciduous and coniferous forests and some wetlands (Butt et al., 2005). Over 1.5 million people live in townships and cities dispersed across the region, with most of the population centred around the city of Ottawa. Mosquito surveillance teams selected 54 sites across Eastern Ontario from areas of historical concern for WNV or EEEV where the viruses had previously infected humans, wild birds or livestock (Figure 1, Table S1).

### Mosquito collections and morphological identifications

Between 2014 and 2019, mosquitoes were collected yearly at each site during the months of June to September using CDC light traps (Drebot et al., 2003). Trapping occurred on a weekly basis except for four sites, which were trapped every 2 weeks (Table S1). CDC light traps baited with a UV lamp and 1.9 kg of dry ice were set at each site prior to 3 pm and collected the next day between 8 am to 12 pm. Traps were set close to dense vegetation at a height of 1 m. After collection, mosquitoes were transported in −20°C coolers to the laboratory, where blood‐fed individuals were separated from individuals without a bloodmeal. These blood‐fed mosquitoes were identified to the species‐level morphologically using a dissecting microscope and custom dichotomous keys (Carpenter, 1965; Means, 1987, 1979; Peyton et al., 1999; Photographic Key to the Adult Female Mosquitoes (Diptera: Culicidae) of Canada, n.d.; Reinert, 2000; Ward, 2005; Wood et al., 1979). *Culex pipiens* and *Culex restuans* were grouped as *Culex pipiens*/*restuans* as distinguishing them morphologically can be unreliable. After identification, mosquitoes were stored dry in freezers (−80°C) and later transported frozen for molecular identification of mosquito species. Only mosquitoes containing macroscopically identifiable undigested bloodmeal were retained in our study and sent to the Canadian Centre for DNA Barcoding in Guelph, Ontario, Canada.

### Molecular identification of mosquitoes and bloodmeal hosts

Mosquitoes were lysed in separate tubes containing 300 μL of lysis buffer, vortexed and incubated overnight at 56°C. DNA was isolated by filtering 50 μL of lysate using glass fibre DNA extraction. PCR was performed on the cytochrome c oxidase (COI) gene region (Alcaide et al., 2009; Reeves et al., 2018), targeting arthropod and bird/mammalian identifier sequences using primers ZBJ‐ArtF1c_t1/ZBJ‐ArtR2_t1 for arthropods (Zeale et al., 2011) and C_BloodmealF1_t1/ModMamRev_t1 for birds and mammals (Reeves et al., 2018). The resulting amplicons were 157 base‐pairs (bp) long for the mosquito arthropod target sequence and 185 bp long for the bird/mammalian target. Each sample was labelled with IonXpress universal molecular identifiers, pooled and sequenced using an Ion Torrent S5 high‐throughput sequencer. Sequencing reads were filtered to a minimum of QV20 quality at least 100 bp in size and trimmed to remove primers and adapters. The remaining reads were referenced to a BOLD library (Ratnasingham & Hebert, 2007) using BLAST (Madden, 2003) to assign taxonomic identities. For taxonomic identification, reads were only accepted if they showed a minimum sequence identity of 95% to a reference sequence over an alignment length of at least 100 bp. In addition, a minimum read count of 100 was required for bird/mammal identification and 500 for mosquitoes. When multiple mosquito species were identified from one sample, the species with the greatest number of supporting reads was used for the sample identity. Even with a minimum sequence identity cutoff of 95%, we recognized that species‐level identifications for host species can be unreliable in many ways (Lou & Golding, 2012; Somervuo et al., 2017) and that this is particularly true when using short molecular markers and when identifying species from a genus with closely related members or owing to an incomplete DNA reference database. To account for this uncertainty, we only considered host species identifications accurate when a potential species was the sole member of its genus in Eastern Ontario; otherwise, we classified the species at the genus level instead (refer to Table S2). For mosquitoes, in cases where sequencing identities conflicted with their morphological identifications, the morphological identity was used.

### Assessing sampling effort

To examine if we captured the full richness of host species fed on by mosquitoes, we generated species accumulation (rarefaction) curves as a function of either sample counts or number of sampling weeks for each mosquito species. These curves can be used to assess if additional sampling effort is needed on a per sample basis and on a temporal basis, respectively. Both plots were generated using R (R Core Team, 2022) and the vegan package (Oksanen et al., 2022) by calculating the mean richness upon each addition of a mosquito sample or sampling week through random permutations of the community dataset. In addition to these plots, we also generated plots of species richness estimates calculated with the Chao estimator of richness using the small sample correction. The Chao estimator is a non‐parametric estimate of minimum species richness and uses the abundance of rare species to predict the number of unobserved species (Chao, 1987). Both plots should reach an asymptote if the full richness of bloodmeal hosts was sampled.

## RESULTS

### Identification of mosquito species

A total of 475 individual mosquito samples with bloodmeals were processed for genetic confirmation of mosquito species and genetic determination of their bloodmeal hosts. From this sample, we were able to genetically assign bloodmeal hosts to the genus or species level for 292 individual mosquitoes (61.5%). Of these 292 mosquitoes, we were able to genetically assign 283 (96.9%) to the species level. For these latter samples, there was direct agreement between morphological and genetic determination of mosquito species for 269 samples (95.1%). Fourteen genetic determinations of mosquito species (4.9%) differed from morphological determination, for which the morphological determination was given precedence (refer to methods). Genetic identification for nine of 292 mosquitoes (3.1%) for which bloodmeal hosts could be genetically determined was not possible and species was assigned based on morphology (refer to methods). One mosquito from the *Ochlerotatus* genus could not be identified to species using either morphology or genetics and was classified as *Ochlerotatus* sp. for our data summaries (Tables 1 and 2, Table S2).

**TABLE 1:** Number of mammal and avian hosts identified in bloodmeals from each mosquito species in Eastern Ontario, Canada.

Mosquito species	Count of mammal hosts	Count of avian hosts
*Aedes cinereus*	13 (100%)	0 (0%)
*Aedes vexans*	112 (99%)	1 (1%)
*Coquillettidia perturbans*	35 (76%)	11 (24%)
*Culex pipiens/restuans*	1 (2%)	42 (98%)
*Ochlerotatus* sp.	1 (100%)	0 (0%)
*Ochlerotatus stimulans gr*.	*16* (100%)	0 (0%)
*Ochlerotatus trivittatus*	79 (99%)	1 (1%)

*Note*: Percentage of total is presented in parentheses after each count. Complete list of genera and species can be found in Table S2.

**TABLE 2:** Summary of mosquito collections with matching bloodmeal host identifications in Eastern Ontario, Canada.

Mosquito species	Total identified	Mean day	Median day	Day range	Year range
*Aedes cinereus*	18	Jul 27	Jul 23	Jun 24–Sep 18	2017–2019
*Aedes vexans*	134	Aug 13	Aug 14	Jun 17–Sep 30	2014–2019
*Coquillettidia perturbans*	51	Jul 27	Jul 26	Jul 01–Sep 27	2017–2019
*Culex pipiens/restuans*	55	Aug 05	Aug 05	Jun 17–Sep 29	2014–2019
*Ochlerotatus* sp.	1	Jul 08	Jul 08	Jul 08–Jul 08	2019–2019
*Ochlerotatus stimulans gr*.	20	Jun 29	Jun 26	Jun17–Aug 08	2017–2019
*Ochlerotatus trivittatus*	91	Aug 10	Aug 10	Jun 26–Sep 27	2014–2019

In total, we were able to genetically identify 313 bloodmeal hosts from our sample of 292 mosquitoes (Table 1, Table S2), with 5.8% of mosquitoes having multiple bloodmeals originating from different species. Of these host identifications, 257 (82.1%) were assigned to the species level given that they represented the sole member of their genus in Ontario at the time of sampling (refer to methods). For the remaining 56 cases (17.9%), hosts were assigned to the genus level. In total, 36 unique hosts were identified, composed of 12 genera and 24 species.

**FIGURE 2:**
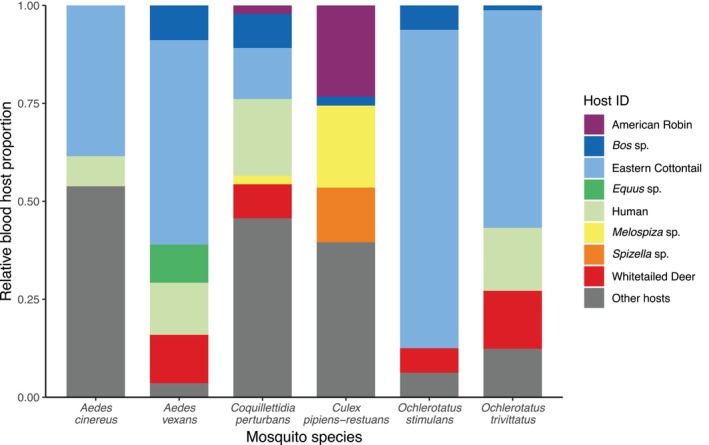
Relative proportion of bloodmeal host species/genera fed on by mosquitoes collected in Eastern Ontario, Canada.

**FIGURE 3:**
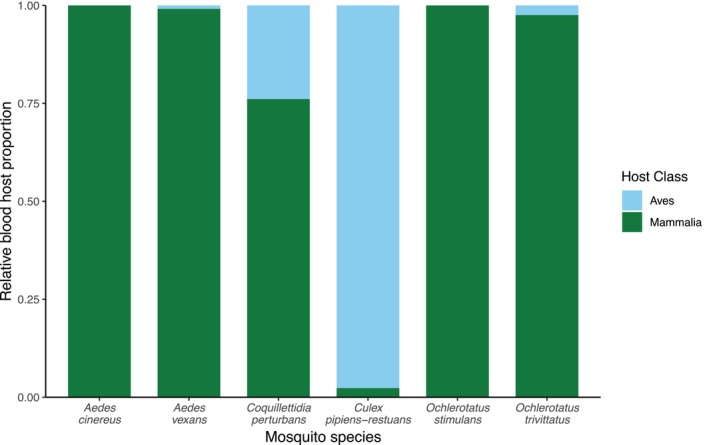
Relative proportion of bloodmeal host classes (birds or mammals) fed on by mosquitoes collected in Eastern Ontario, Canada.

Bloodmeal host composition varied across mosquito species and genera (Table 1, Table S2); 98% of *C. pipiens/restuans* bloodmeal hosts were birds, with the majority being composed of American robins (23.3%, *Turdus migratorius*), followed by species belonging to the genera *Melospiza* (20.9%) and *Spizella* (14.0%; Figures 2 and 3). *C. perturbans* fed on mammals (76.1%) and birds (23.9%) from a variety of species and genera, with the largest percentages from humans (19.6%), eastern cottontail rabbits (*Sylvilagus floridanus*; 13.0%) and eastern grey squirrels (*Sciurus carolinensis*; 10.9%). *Aedes* and *Ochlerotatus* species mainly fed on mammals (99.3% and 98.0%, respectively). For the *Aedes* genus, the most common bloodmeals were from eastern cotton tail rabbits (56.6%), humans (14.2%) and white‐tailed deer (*Odocoileus virginianus*; 12.4%). For the *Ochlerotatus* genus, the most common bloodmeals were from eastern cottontail rabbits (59.2%), humans (14.3%) and white‐tailed deer (13.3%). Across all mosquito species, eastern cottontail rabbits accounted for 41% of all bloodmeal identifications.

### Sampling effort of bloodmeal host community

Sampling effort within and across years for mosquito species was relatively consistent, with *A. vexans*, *Ochlerotatus trivittatus* and *C. pipiens/restuans* surveyed between June to September each year from 2014 to 2019 (Table 2). *C. perturbans* differed from the other species in only having been sampled from 2017 to 2019, although monthly coverage was similar to other species.

**FIGURE 4:**
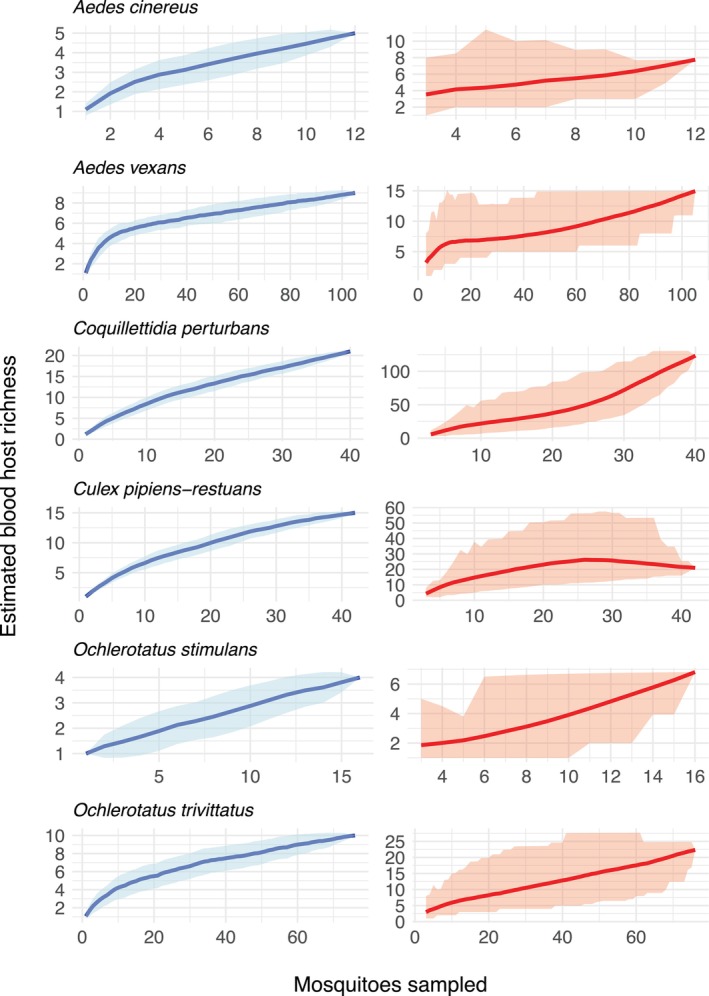
Species rarefaction curves (left) and Chao estimator (right) for bloodmeal host species richness and sampled mosquitoes in Eastern Ontario, Canada. Shaded areas represent 95% confidence intervals for fitted line.

**FIGURE 5:**
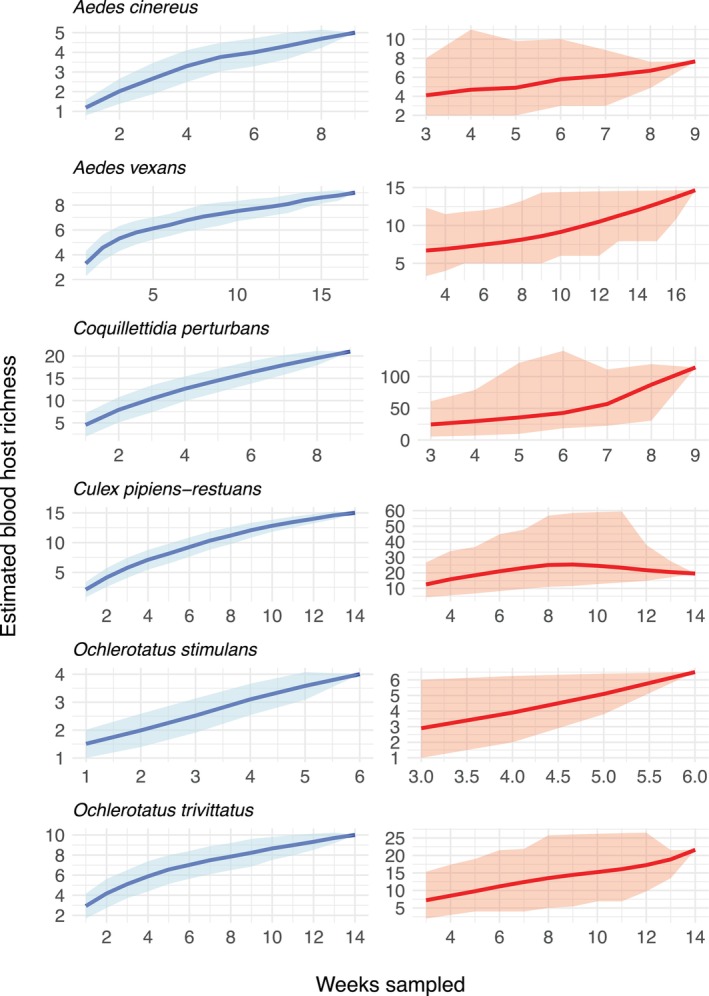
Species rarefaction curves (left) and Chao estimator (right) for bloodmeal host species and sampled weeks in Eastern Ontario, Canada. Shaded areas represent 95% confidence intervals for fitted line.

Neither the rarefaction or Chao estimator curves reached a stable asymptote when considering either sample size or number of sampling weeks (Figures 4 and 5, respectively). This indicates that a greater effort will be needed to fully understand the diversity of bloodmeal hosts utilized by mosquito species involved in pathogen transmission in our study area.

## DISCUSSION

In this study we identified bloodmeal hosts used by mosquitoes over a broad region in Eastern Ontario, Canada. The most commonly sampled mosquitoes with bloodmeals included *A. vexans*, *O. trivittatus, C. pipiens/restuans* and *C. perturbans* mosquitoes that are capable of or have been implicated in transmitting WNV to some extent (Sardelis, 2001; Tiawsirisup et al., 2008; Tiawsirisup, Platt, Evans, & Rowley, 2005; Turell et al., 2001) and only likely serve a minor role in EEEV transmission (Armstrong & Andreadis, 2010; Cupp et al., 2003; Turell, 2012; Vaidyanathan et al., 1997). The exception to the latter is *C. perturbans*, which is potentially a bridge vector for EEEV (Armstrong & Andreadis, 2022; but refer to Sardelis, 2001; Turell et al., 2001). *Aedes* and *Ochlerotatus* mosquitoes had more bloodmeals from mammals than birds, *C. pipiens/restuans* had more bloodmeals from birds than mammals and *C. perturbans* had bloodmeals from comparable numbers of birds and mammals. For the mosquitoes feeding primarily on mammals, the *Aedes* and *Ochlerotatus* mosquitoes, eastern cottontails were always the most common sources identified in bloodmeals. Americans robins (*Turdus migratorius*), a strong amplification host for WNV and EEEV (Komar et al., 1999, 2003), was the most common bird species fed on by *C. pipiens/restuans*, highlighting a potential mechanism by which WNV enzootic cycles are maintained in our study region. American robins and humans were also fed on by *C. perturbans*, a potential bridge vector for EEEV, illustrating a hypothetical transmission pathway in our system. Last, while *A. vexans*, a potential bridge vector for WNV, was not observed to forage on known amplifying hosts, it was observed to forage on humans at a relatively high frequency, highlighting a potential exposure pathway of humans to WNV. Together, these results suggest that habitats where these mosquitoes and birds commonly occur, such as well‐vegetated residential areas (Clergeau et al., 1998; Vanderhoff et al., 2016), may see increased WNV and EEEV transmission risk to humans.

Our initial observations of mosquitoes having more bloodmeals from certain animal groups are generally consistent with patterns found in the literature for different regions. Other studies regularly show greater mammalian feedings for *A. vexans* (Börstler et al., 2016; Greenberg et al., 2013; Hamer et al., 2009; Molaei & Andreadis, 2006; Nasci, 1984), *A. cinereus* (Apperson et al., 2004; Molaei et al., 2008), *O. sollicitans* (Apperson et al., 2004; Molaei et al., 2008) and *O. trivittatus* (Hamer et al., 2009; Molaei et al., 2008; Nasci, 1984), while *C. pipiens/restuans* tend to prefer feeding on bird species (Apperson et al., 2002; Hamer et al., 2009; Khalil et al., 2021; Kothera et al., 2020; Molaei et al., 2006; Taieb et al., 2020). *C. perturbans* mosquitoes appeared to feed on more mammals than birds, which has been observed previously (Anderson et al., 2018; Gingrich & Williams, 2005; Molaei et al., 2008). Although these patterns may be typical for these mosquitoes, exceptions do exist, further highlighting the need for monitoring and research at a regional level. In Germany for example, *C. pipiens* mosquitoes were observed feeding on more mammals than birds (Börstler et al., 2016). In the US, mosquito species were observed feeding on different proportions of mammals and birds depending on the region (Apperson et al., 2004), with *C. pipiens* and *C. restuans* utilizing more mammalian bloodmeals in New Jersey compared to New York. These studies, among others (Chaves et al., 2010; Lyimo & Ferguson, 2009; Wolff & Riffell, 2018), show that although mosquitoes have tendencies to feed on certain animal groups, local bloodmeal host availability is a strong determining factor of mosquito diets. Other factors like hybridization between mosquitoes and local adaptations of mosquito populations, may also contribute to mosquito diets that are drastically different between regions (Fonseca et al., 2004).

Based on our data, we suggest a transmission cycle for WNV could exist in our study area between *Culex* species and American robins and that *A. vexans* could play a role in human exposure. Specifically, we observed a relatively high frequency of bloodmeals from American robins for *Culex* species. American robins are relatively abundant in our study region and are well‐known ‘super spreaders’ for WNV elsewhere (Hamer et al., 2009; Kilpatrick, Daszak, et al., 2006). We also observed *A. vexans*, a potential bridge vector for WNV (Tiawsirisup et al., 2008; Tiawsirisup, Platt, Evans, & Rowley, 2005; Turell et al., 2001), foraging on a relatively high frequency of humans. Again, given American robins' affinity for vegetated areas in urban ecosystems (Clergeau et al., 1998; Vanderhoff et al., 2016), combined with a relatively high abundance of *Culex* species and *A. vexans* in sub‐urban ecosystems in our study region (Cloutier et al., 2021), there is high potential for spread to humans through amplification of viral prevalence and the presence of bridge vectors in these areas. It has also been suggested that *C. pipiens* can function both as an enzootic vector and bridge vector of WNV, even with primarily avian diets, due to its high relative abundance and tendency to occasionally feed on humans (Hamer et al., 2008; Kilpatrick et al., 2005). It is unclear however if this is the case in our region given that we did not detect any human bloodmeals from *C. pipiens*, which could be representative of their local diets or a result of limited detection power from a smaller sample size. Our data also suggests a possible transmission pathway by which humans could become exposed to EEEV in our study region. While we did not capture any *Culiseta melanura*, the primary vector for EEEV (Armstrong & Andreadis, 2010), with bloodmeals in this study, *C. perturbans* was observed to have fed on an American Robins, an amplification host (Armstrong & Andreadis, 2022) and a relatively high frequency of humans. Similar to *A. vexans* for WNV, this mosquito could function as a critical ‘bridge vector’ between infected birds and humans in this region for EEEV, as has been suggested for other regions in North America (Armstrong & Andreadis, 2010; Armstrong & Andreadis, 2022). We caution that these transmission cycles and exposure pathways are hypothetical. To be certain that these pathways exist, we would require a better understanding of local mosquitoes' vectorial capacity, viral prevalence in bloodmeal hosts, evidence for significant transmission, and whether observations are due to bloodmeal host preference or host availability.

We observed a high proportion of bloodmeals from various mammals and notably, eastern cottontail rabbits, which have been shown to potentially play a role in WNV transmission in laboratory settings (Tiawsirisup, Platt, Tucker, & Rowley, 2005). However, in North America, the natural pathogen transmission cycles of WNV and EEEV are believed to be predominantly between birds and mosquitoes, with occasional transmission to dead‐end hosts like horses and humans (Davis et al., 2008; Dugassa et al., 2016; Guo et al., 2022; Hajibabaei et al., 2006; Martin et al., 2019; Reeves et al., 2016). In general, mammals have consistently shown little or no competence for WNV or EEEV (Root, 2013; Root & Bosco‐Lauth, 2019), although exceptions like eastern chipmunks (Platt et al., 2007) and eastern cottontails (Tiawsirisup, Platt, Tucker, & Rowley, 2005) show that generalizations should be made cautiously. However, given the demonstrated ability for eastern cottontail rabbits to transmit WNV in a laboratory setting (Tiawsirisup, Platt, Tucker, & Rowley, 2005) and their abundance in the wild, we suggest that targeted WNV surveillance in wild eastern cottontail rabbits would more definitely rule out their role in WNV transmission cycles in eastern North America.

An important caveat to our study is that based on our species accumulation and rarefaction curves failing to plateau, we did not capture the entire diversity of bloodmeal hosts of mosquitoes in our study region. As such, the diets of mosquitoes that we describe are not fully representative of the diets for each species and we could be missing important amplification or dilution hosts involved in these transmission dynamics. One reason we were unlikely to have captured their diet completely is that our sampling was opportunistic, in that blood‐fed mosquitoes were subsampled from a larger sampling effort for mosquito diversity and abundance across a wide region. Had we used traps specifically designed for gravid females (Dugassa et al., 2016), we may have been able to acquire more bloodmeals, although given logistic constraints it would have been challenging to have surveyed an equally large area. Another reason we were unable to sample the entire diet breadth of mosquitoes was that despite our best efforts we only had positive bloodmeal identifications for 61.5% of all mosquito specimens examined. This is likely a function of degradation of DNA owing to digestion by the mosquitoes prior to collection and degradation of the DNA from freezing (Reeves et al., 2016), when samples were stored both in the field and after manual identification. To account for this, we used markers, that is, mini‐ or minimalist barcodes (Dugassa et al., 2016; Reeves et al., 2016) which can sometimes not distinguish species from the same genus. This is part of the reason we only assigned species‐level identifications when the species in question was the sole species belonging to that genus at the time of sampling. Nonetheless, we recommend future research increases sample sizes by specifically targeting gravid females and storing bloodmeals on filter paper, which best preserves DNA for barcoding (Reeves et al., 2016).

Another important caveat to our study is that we did not assess host competence: the ability of an infected host to transmit the infection to another (Ciota & Kramer, 2013; Martin et al., 2019). If the animal species fed on by mosquitoes are susceptible to dying quickly to the viral infection, they may not survive long enough to re‐infect other mosquitoes. Similarly, if the viral infection does not result in a strong viral density in blood (i.e., the viral load), feeding mosquitoes are unlikely to be infected and these animals can be considered ‘dead‐end’ hosts (Kramer et al., 2008; Reisen, 2013). Animal species that produce higher viral loads from infection and readily infect feeding mosquitoes, are considered competent hosts. In an ecological context, if mosquitoes are feeding primarily on dead‐end hosts, these animal species can function as dilution hosts that decrease the overall prevalence of pathogens in their environment. If mosquitoes are feeding primarily on competent species, these animals can also be considered amplification hosts if they increase the overall pathogen prevalence in their area. Animals that have higher viral loads from infection, carry these infections for a long period and are generally unaffected by the infection, can be additionally classified as reservoirs hosts for these pathogens. For WNV and EEEV, mammals, reptiles, amphibians and birds respond very differently to infection from these viruses (Pérez‐Ramírez et al., 2014; Rissmann et al., 2020; Root, 2013; Root & Bosco‐Lauth, 2019). Even within these groups, each species also shows significant variation in their viral loads from infection and their ability to transmit the virus to feeding mosquitoes. For WNV and EEEV, certain bird species are consistently implicated as the primary reservoir and amplification hosts, although many bird species can reach viral loads capable of infecting feeding mosquitoes (Burkett‐Cadena et al., 2022; Komar et al., 2003; Pérez‐Ramírez et al., 2014). Compared to avian hosts, the competence of mammalian and reptilian hosts is understudied, but a few species may be capable of transmitting the virus (Klenk et al., 2004; Rissmann et al., 2020; Root, 2013; Root & Bosco‐Lauth, 2019). It is unclear currently how each bloodmeal host identified in this study would contribute to the overall amplification or dilution of these arboviruses, and we encourage more work in this area to understand the role each of these taxonomic groups has in arboviral dynamics locally.

## CONCLUSION

Our results provide the first observations of the bloodmeal host community for mosquito species in Eastern Ontario, Canada, and support observations from the literature with respect to individual mosquito species and their general preferences for mammals versus birds. We identified possible mechanisms by which *Culex* species in our region may maintain a WNV transmission cycle and how *A. vexans* and *C. perturbans* could be acting as bridge‐vectors to humans for WNV and EEEV, respectively. Furthermore, our sampling was opportunistic and the lack of asymptotes in our species accumulation and rarefaction curves illustrate a general need for more focused and additional sampling in the future to more fully characterize mosquito bloodmeal hosts. For example, traps specifically designed to catch gravid females (Dugassa et al., 2016) would likely provide a more complete description of bloodmeal hosts in our study region and storing bloodmeals on filter paper would better preserve DNA (Reeves et al., 2016). Going forward, it will be important to continue monitoring mosquito abundances, host preferences, host abundances and WNV and EEEV prevalence through time, given that climate change is predicted to increase pathogen prevalence and mosquito abundance (Hongoh et al., 2012; Ludwig et al., 2019; Ogden et al., 2019), as well as host abundance (Viana & Chase, 2022), in temperate northern regions.

## AUTHOR CONTRIBUTIONS

**Colton R. A. Stephens:** Conceptualization; investigation; writing – original draft; methodology; validation; visualization; writing – review and editing; software; formal analysis; project administration; data curation; resources. **Mark R. Forbes:** Conceptualization; supervision; funding acquisition; writing – review and editing; methodology; project administration; resources; validation. **Scott Wilson:** Funding acquisition; writing – review and editing; methodology; conceptualization; resources; project administration; data curation. **Antoinette Ludwig:** Writing – review and editing; methodology; conceptualization; resources; project administration; data curation; funding acquisition. **David Lapen:** Funding acquisition; writing – review and editing; resources; project administration; conceptualization; methodology; data curation. **Greg W. Mitchell:** Conceptualization; methodology; software; data curation; supervision; formal analysis; validation; investigation; funding acquisition; visualization; project administration; resources; writing – review and editing.

## FUNDING INFORMATION

This work was supported by funding provided by Agriculture and Agri‐Food Canada's Environmental Change Onehealth Observatory ECO2 (https://agriculture.canada.ca/en; GM, AL, DL): grant J‐002305.001.07. Additional funding was provided by Environment and Climate Change Canada (https://www.canada.ca/en/environment-climate-change.html; GM, SW), Agriculture and Agri‐Food Canada (https://agriculture.canada.ca/en; DL), The Public Health Agency of Canada (https://www.canada.ca/en/public-health.html; AL) and Natural Sciences and Engineering Research Council of Canada NSERC Discovery Grant 100118 (https://www.nserc-crsng.gc.ca/index_eng.asp; MF).

## CONFLICT OF INTEREST STATEMENT

The authors declare no conflicts of interest.

## Supporting information


**Table S1.** Description of mosquito trapping sites and trapping schedule in Eastern Ontario, Canada.
**Table S2**. Identified bloodmeal hosts sampled from mosquito species in Eastern Ontario. Please note that the *Bos* and Equus genera are domestic in our research area, that *Canus* can refer to domestic dogs or wild coyotes and that Sus most likely refers to domestic swine, but could also represent wild pigs.

## Data Availability

Data used in this manuscript are openly available from the Open Science Framework Repository https://doi.org/10.17605/OSF.IO/K4SY6 (Stephens et al., 2026).
