# Supplementary material for: Mosquito feeding patterns in the context of West Nile and eastern equine encephalitis viruses in eastern Ontario, Canada
Source: Med Vet Entomol. 2026 May 14;40(3):634–46. doi: 10.1111/mve.70077 (PMC13436511; doi:10.1111/mve.70077)
Supplement: Supplementary file 1 [file MVE-40-634-s001.docx]

## 1. Supporting information

Supplemental tables

Table S1. Description of mosquito trapping sites and trapping schedule in Eastern Ontario, Canada.

| **Site** | **Lat** | **Long** | **Years Sampled** | | **Trapping Schedule** |
| --- | --- | --- | --- | --- | --- |
| AL 110A | 45.22870 | -76.18920 | 2018 | Weekly | |
| AX2 | 45.31467 | -74.64369 | 2017 | Bi-weekly | |
| CA3 | 45.31435 | -75.08413 | 2018 - 2019 | Bi-weekly | |
| CO12 | 45.03125 | -74.71414 | 2017 - 2019 | Weekly | |
| CO20 | 45.01653 | -74.76681 | 2017 | Weekly | |
| CP 110A | 45.13180 | -76.01590 | 2019 | Weekly | |
| CR1 | 45.21888 | -75.15638 | 2018 | Bi-weekly | |
| EM3 | 45.26412 | -75.27905 | 2017 | Weekly | |
| H3 | 45.60775 | -74.58836 | 2017 | Bi-weekly | |
| OTW001 | 45.36540 | -75.62310 | 2014 - 2019 | Weekly | |
| OTW005 | 45.48410 | -75.47380 | 2015 - 2019 | Weekly | |
| OTW006 | 45.34710 | -75.63450 | 2014 - 2019 | Weekly | |
| OTW007 | 45.34810 | -75.66600 | 2017 - 2019 | Weekly | |
| OTW009 | 45.28400 | -75.76260 | 2014 | Weekly | |
| OTW010 | 45.32760 | -75.77470 | 2017 - 2018 | Weekly | |
| OTW011 | 45.38600 | -75.72770 | 2014 - 2019 | Weekly | |
| OTW013 | 45.31930 | -75.89120 | 2017 - 2019 | Weekly | |
| OTW014 | 45.29600 | -75.87760 | 2017 - 2019 | Weekly | |
| OTW015 | 45.27591 | -75.91684 | 2017 - 2019 | Weekly | |
| OTW016 | 45.34070 | -75.97960 | 2014 - 2019 | Weekly | |
| OTW019 | 45.32069 | -75.82215 | 2017 - 2019 | Weekly | |
| OTW020 | 45.21960 | -75.67840 | 2014 - 2019 | Weekly | |
| OTW021 | 45.23600 | -75.56150 | 2017 | Weekly | |
| OTW022 | 45.23200 | -75.47520 | 2017 | Weekly | |
| OTW023 | 45.32779 | -75.44434 | 2019 | Weekly | |
| OTW024 | 45.42610 | -75.40140 | 2014 - 2019 | Weekly | |
| OTW025 | 45.51710 | -75.41030 | 2017 - 2018 | Weekly | |
| OTW309 | 45.35560 | -75.70460 | 2017 - 2019 | Weekly | |
| OTW313 | 45.41214 | -75.71005 | 2019 | Weekly | |
| OTW500 | 45.43750 | -75.64550 | 2017 - 2019 | Weekly | |
| OTW502 | 45.40910 | -75.64960 | 2014 - 2019 | Weekly | |
| OTW505 | 45.46078 | -75.50411 | 2018 - 2019 | Weekly | |
| OTW507 | 45.24910 | -75.91833 | 2014 - 2019 | Weekly | |
| OTW508 | 45.27420 | -75.76340 | 2018 | Weekly | |
| OTW509 | 45.33090 | -75.92070 | 2014 - 2019 | Weekly | |
| OTW510 | 45.24724 | -75.72377 | 2017 - 2018 | Weekly | |
| OTW511 | 45.45510 | -75.48188 | 2017 - 2019 | Weekly | |
| OTW512 | 45.50025 | -76.09263 | 2017 - 2019 | Weekly | |
| OTW513 | 45.34992 | -75.83311 | 2017 - 2019 | Weekly | |
| OTW514 | 45.43979 | -75.55982 | 2017 - 2019 | Weekly | |
| OTW515 | 45.45972 | -75.61443 | 2018 - 2019 | Weekly | |
| OTW517 | 45.31411 | -75.59821 | 2019 | Weekly | |
| PE 110A | 44.88983 | -76.23970 | 2017 - 2018 | Weekly | |
| PR 14A | 44.71910 | -75.51910 | 2017 | Weekly | |
| R02 | 45.53762 | -75.29099 | 2017 | Weekly | |
| S11P3 | 45.26111 | -75.55909 | 2018 | Weekly | |
| S13P2 | 45.31503 | -75.60954 | 2018 | Weekly | |
| S13P4 | 45.31380 | -75.60736 | 2018 | Weekly | |
| S2P4 | 45.27241 | -75.16571 | 2018 | Weekly | |
| S3P4 | 45.28148 | -75.15457 | 2018 | Weekly | |
| S6P2 | 45.27862 | -75.22250 | 2018 | Weekly | |
| S7P3 | 45.28683 | -75.22733 | 2018 | Weekly | |
| S8P3 | 45.21981 | -75.15142 | 2018 | Weekly | |
| SF 110A | 44.89390 | -76.01593 | 2017 - 2019 | Weekly | |

Table S2. Identified bloodmeal hosts sampled from mosquito species in Eastern Ontario. Please note that the *Bos* and Equus genera are domestic in our research area, that *Canus* can refer to domestic dogs or wild coyotes, and that Sus most likely refers to domestic swine, but could also represent wild pigs.

| **Mosquito Species** | | **Bloodmeal Host** | | **Count** |
| --- | --- | --- | --- | --- |
| ***Aedes cinereus*** |  | |  | |
|  | | Human (*Homo sapiens*) | | 1 (7.7%) |
|  | | *Microtus* | | 1 (7.7%) |
|  | | Eastern chipmunk (*Tamias striatus*) | | 5 (38.5%) |
|  | | Eastern cottontail (*Sylvilagus floridanus*) | | 5 (38.5%) |
|  | | Eastern gray squirrel (*Sciurus carolinensis*) | | 1 (7.7%) |
| Total hosts | |  | | 13 |
| ***Aedes vexans*** |  | |  | |
|  | | Human | | 15 (13.3%) |
|  | | Raccoon (*Procyon lotor*) | | 1 (0.9%) |
|  | | *Bos* | | 10 (8.8%) |
|  | | *Canis* | | 1 (0.9%) |
|  | | *Equus* | | 11 (9.7%) |
|  | | *Sus* | | 1 (0.9%) |
|  | | White-tailed deer (*Odocoileus virginianus*) | | 14 (12.4%) |
|  | | Eastern cottontail | | 59 (52.2%) |
|  | | House sparrow (*Passer domesticus*) | | 1 (0.9%) |
| Total hosts | |  | | 113 |
| ***Coquillettidia perturbans*** |  | |  | |
|  | | Human | | 9 (19.6%) |
|  | | Raccoon | | 3 (6.5%) |
|  | | American red squirrel (*Tamiasciurus hudsonicus*) | | 1 (2.2%) |
|  | | *Anas* | | 1 (2.2%) |
|  | | Baltimore oriole (*Icterus galbula*) | | 1 (2.2%) |
|  | | *Bos* | | 4 (8.7%) |
|  | | *Canis* | | 1 (2.2%) |
|  | | Cedar waxwing (*Bombycilla cedrorum*) | | 1 (2.2%) |
|  | | *Falco* | | 1 (2.2%) |
|  | | *Melospiza* | | 1 (2.2%) |
|  | | Muskrat (*Ondatra zibethicus*) | | 1 (2.2%) |
|  | | Rosebreasted grosbeak (*Pheucticus ludovicianus*) | | 1 (2.2%) |
|  | | *Spinus* | | 1 (2.2%) |
|  | | *Sus* | | 1 (2.2%) |
|  | | White-tailed deer | | 4 (8.7%) |
|  | | Blue jay (*Cyanocitta cristata*) | | 1 (2.2%) |
|  | | Eastern cottontail | | 6 (13.0%) |
|  | | Eastern gray squirrel | | 5 (10.9%) |
|  | | American robin (*Turdus migratorius*) | | 1 (2.2%) |
|  | | European starling (*Sturnus vulgaris*) | | 1 (2.2%) |
|  | | House sparrow | | 1 (2.2%) |
| Total hosts | |  | | 46 |
| ***Culex pipiens/restuans*** |  | |  | |
|  | | Black-capped chickadee (*Poecile atricapillus*) | | 2 (4.7%) |
|  | | *Bos* | | 1 (2.3%) |
|  | | Cedar waxwing | | 2 (4.7%) |
|  | | Green heron (*Butorides virescens*) | | 1 (2.3%) |
|  | | *Melospiza* | | 9 (20.9%) |
|  | | Mourning dove (*Zenaida macroura*) | | 2 (4.7%) |
|  | | *Setophaga* | | 1 (2.3%) |
|  | | *Sitta* | | 1 (2.3%) |
|  | | *Spizella* | | 6 (14.0%) |
|  | | Wild turkey (*Meleagris gallopavo*) | | 1 (2.3%) |
|  | | Blue jay | | 1 (2.3%) |
|  | | American Robin | | 10 (23.3%) |
|  | | Gray catbird (*Dumetella carolinensis*) | | 1 (2.3%) |
|  | | House sparrow | | 2 (4.7%) |
|  | | Northern cardinal (*Cardinalis cardinalis*) | | 3 (7.0%) |
| Total hosts | |  | | 43 |
| ***Ochlerotatus sp.*** |  | |  | |
|  | | Human | | 1 (100.0%) |
| Total bloodmeal hosts | |  | | 1 |
| ***Ochlerotatus stimulans gr.*** |  | |  | |
|  | | *Bos* | | 1 (6.2%) |
|  | | *Canis* | | 1 (6.2%) |
|  | | White-tailed deer | | 1 (6.2%) |
|  | | Eastern cottontail | | 13 (81.2%) |
| Total hosts | |  | | 16 |
| ***Ochlerotatus trivittatus*** |  | |  | |
|  | | Human | | 13 (16.0%) |
|  | | Domestic cat (*Felis catus*) | | 2 (2.5%) |
|  | | Raccoon | | 4 (4.9%) |
|  | | *Bos* | | 1 (1.2%) |
|  | | Pileated woodpecker (*Dryocopus pileatus*) | | 1 (1.2%) |
|  | | *Sus* | | 1 (1.2%) |
|  | | Water buffalo (*Bubalus bubalis*) | | 1 (1.2%) |
|  | | White-tailed deer | | 12 (14.8%) |
|  | | Wild Turkey | | 1 (1.2%) |
|  | | Eastern cottontail | | 45 (55.6%) |
| Total hosts | |  | | 81 |
